# The Effect of Traditional and Non-Thermal Treatments on the Bioactive Compounds and Sugars Content of Red Bell Pepper

**DOI:** 10.3390/molecules25184287

**Published:** 2020-09-18

**Authors:** Katarzyna Rybak, Artur Wiktor, Dorota Witrowa-Rajchert, Oleksii Parniakov, Małgorzata Nowacka

**Affiliations:** 1 Department of Food Engineering and Process Management, Institute of Food Sciences, Warsaw University of Life Sciences–SGGW, 02-787 Warsaw, Poland; katarzyna_rybak@sggw.edu.pl (K.R.); artur_wiktor@sggw.edu.pl (A.W.); dorota_witrowa_rajchert@sggw.edu.pl (D.W.-R.); 2 Elea Vertriebs- und Vermarktungsgesellschaft mbH, 49610 Quakenbrück, Germany; o.parniakov@elea-technology.com

**Keywords:** red bell pepper, pre-treatment, blanching, ultrasounds treatment, pulsed electric field treatment, combined treatment, bioactive compounds

## Abstract

The aim of the study was an investigation of the effect of traditional and non-thermal treatment on the bioactive compounds of red bell pepper. As a thermal process, blanching in water and in steam was studied, while for non-thermal the sonication, pulsed electric field treatment and their combination were used in this experiment. The red bell peppers were evaluated based on quality attributes such as: total carotenoids content; polyphenols; vitamin C; antioxidant activity and sugars content. Vitamin C and sugar content were analyzed using liquid chromatography and other measurements were determined based on the spectrophotometric method. Results showed that the blanching in water or in steam reduced bioactive compounds concentration; whereas non-thermal treatments as pulsed electric field (PEF) applied separately or in combination with ultrasound (US + PEF) let to obtain similar or slightly lower content of bioactive compounds in comparison to untreated peppers. When sonication (US) and combined treatment as PEF + US were applied; in most cases reduction of bioactive compounds concentration occurred. This effect was probably related to the effect of relatively long (30 min) ultrasound treatment. The application of appropriate parameters of non-thermal processing is crucial for the high quality of processed material.

## 1. Introduction

Red bell pepper (*Capsicum annuum* L.) is characterized by intensive red color, strong and/or sweet flavor, crispy texture and thin wax layer [1,2]. This well-known vegetable is also valued by consumers for its high content of bioactive substances such as carotenoids, flavonoids, polyphenols, vitamins C, A, E, B_1_, B_2_ [3,4,5,6]. However, after harvesting, due to its high water content, red bell pepper is very sensitive for microbiological activity and thus spoilage. Moreover, in a ripe vegetable occurs many different enzymatic reactions, which may cause degradation of product quality [5]. Therefore the shelf-life of pepper even in cold room storage is quite short. In this regard, various methods of processing of the bell pepper such as blanching, freezing [7], pickling [4,8], osmotic dehydration [9,10] and drying [11,12,13] are proposed to, leading to extend the shelf life.

Blanching is a traditional pre-treatment that is used to inactivate enzymes such as peroxidase, polyphenol oxidase and pectinase. This thermal treatment leads to inhibition of browning reaction and color changes of plant tissue, reduces the microbial load to obtain longer shelf life and removes air from the tissue that affects positively mass and heat transfer [3,5,14]. However, after blanching also undesirable effects are observed such as changes in sensory profile, texture and quality due to reducing nutrients and vitamins [15]. This operation is usually conducted in hot water due to its simplicity [16]. However other blanching methods recently have been studied. These methods limit the negative effects of this operation. For example, blanching with steam instead of hot water minimizes leaching of water-soluble compounds to the surroundings and higher content of heat-sensitive compounds is preserved [17]. Similarly, to replace hot water blanching, the high-humidity hot air impingement blanching (HHAIB) was proposed by Wang et al. [14]. This method results in rapid blanching what minimizes nutrient loss. Also, the infrared and microwave blanching can be used and usually better color, higher vitamin C retention and higher antioxidant activity of plant material are gained in comparison to traditional blanching in water [14]. This can be explained by formation of derivatives of phenolic compounds that improves antioxidant activity [5]. This operation is applied before such processes as cutting, peeling, freezing [5], osmotic dehydration or drying [18].

Ultrasound (US) is one of the non-thermal technologies which can be used for inactivation or activation of the enzymes, cleaning, decontamination of the surface, enhancement of extraction, osmotic dehydration, freezing, drying, etc. [18,19]. The US treatment shows promising features as a method supporting food production processes. However its effect depends on the type of raw material, its structure and anatomy, as well as properties of applied sound waves (length, frequency, intensity, attenuation coefficient) and duration of application [20,21,22]. According to Sledz et al. [23] specific parameters of ultrasound treatment applied to parsley leaves before microwave drying intensified the drying process by 29.8% whereas steam blanching reduced the drying time by 17.5%. Also, the US treatment increases the chlorophyll retention in the dried parsley in comparison to steam blanching. However, the stability of the color of dried parsley leaves increases for both blanched and sonicated samples compared to the material without treatment. It is well known, that US impacts the physical and chemical properties and the structure of final products due to the “sponge effect” and cavitation effects which lead to the microchannel formation [24,25].

Pulsed electric field (PEF) is considered as one of the most promising treatment methods which cause reversible or irreversible changes of the cell membrane. Due to the electroporation, during application of PEF the continuity of cell membrane might be disrupted and this positively affects mass and heat transfer based processes and reduces microbial load [26,27,28]. For example, the PEF treatment of bell pepper resulted in the intensification of mass transfer during osmotic dehydration. Water loss was 11–25% higher whereas solid gain remained almost unchanged in comparison to untreated material, respectively [9]. According to the literature, the PEF application influence the sensory profile of products such as color, taste or flavor and also allows the preservation of higher content of pro-healthy and functional compounds [26].

Generally, the pre-treatment, applied by traditional or non-thermal methods, can performed to obtain ready to eat minimally processed product or to precede proper processing such as drying, freezing, etc. [18,29]. In recent years, the combination of different pretreatment with each other and with other processes is reported in the scientific publications in order to obtain specific purposes and to keep the high quality of the product [30]. For example, the combination of thermal and non-thermal processes such as blanching in water, PEF treatment with sonication, which was subjected to osmotic dehydration and hot-air drying of cranberries allowed to reduce the drying time in the range from 48 to 55% in comparison to traditionally treated material [18]. Tao et al. [31] enhanced the convective drying and reduced the process up to 83.4% by applying blanching coupled with contact sonication. Moreover, the Salas-Tovar et al. [8] applied the ultrasound-assisted vacuum-infusion with a solution of CaCl_2_ and pectin methylesterase and found that blanching-induced changes in color and texture of jalapeño pepper were reduced compared to untreated sample.

Therefore, it is necessary to continue the research, which would help better understanding the effects occurring during the application of pre-treatments and their impact on the properties of food products. Thus, in the study, the aim was to determine the effect of traditional/thermal, non-thermal and combined pre-treatment methods on the bioactive compounds of red bell pepper. To analysis, the influence treatment on bioactive compounds such as total carotenoids content, polyphenols, vitamin C, the antioxidant activity was evaluated. Also, the effect of traditional and non-thermal treatment on the sugars content of red bell pepper was assessed.

## 2. Results and Discussion

### 2.1. Effect of Traditional and Non-Thermal Treatment on the Total Carotenoid Content of Red Bell Peppers

**Figure 1:**
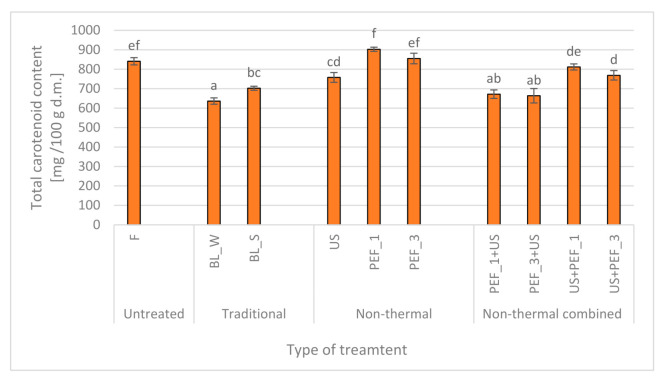
Total carotenoid content in fresh (F) red bell peppers and subjected to traditional (blanching in water, BL_W, and blanching in steam, BL_S) and non-thermal separate (US—ultrasound, PEF_1 and PEF_3—pulsed electric field with energy input 1 and 3 kJ/kg, respectively) and combined treatment (PEF + US and US + PEF), the same letters indicate homogeneous groups (Tukey’s HSD, *p* < 0.05), the error bars represent the standard deviation calculated from 3 repetitions.

Blanching, regardless of the utilized method, lead to the significant (*p* < 0.05) reduction of carotenoids (Figure 1). Samples blanched in steam demonstrated total carotenoid content of 702 mg/100 g dry matter (d.m.) whereas the material treated by hot water exhibited even lower carotenoid content which was equal to 636 mg/100 g d.m. It means that traditional treatment reduced the concentration of carotenoid by 16.5 and 24.3% in the case of water and steam blanching method, respectively. Carotenoids are sensitive for oxidation and hence, elevated temperature of hot water or steam blanching accelerated their degradation during treatment. It was previously demonstrated that thermal degradation of carotenoids in papaya puree follows the first order reaction kinetics [32]. The difference between hot water and steam blanching may be related to the specificity of treatment—water that penetrates disintegrated tissue may flush out carotenoid molecules. What more, although most of carotenoids are water insoluble they can form complexes with proteins which affects their polarity and thus water solubility [33]. Also, some of carotenoids exhibit higher polarity than others—most of xanthophyll’s are more polar than other carotenoids [34]. This explanation fits to the results achieved by application of ultrasound. The concentration of carotenoids in sonicated (US) samples was equal to 758 mg/100 g d.m. This value was significantly higher than the one obtained for hot water blanching and similar, from statistical point of view, that was achieved for blanched material. In turn, PEF treated material was characterized by total carotenoid content of 903 and 855 mg/100 g d.m., when 1 and 3 kJ/kg of energy input was applied, respectively. Such results are most probably associated with electroporation phenomenon and thus better extractability of carotenoid from disintegrated tissue. What is interesting, smaller content of carotenoids was found in sample treated by higher energy input (3 kJ/kg). Similar reports were previously presented in the literature for carrot PEF treatment [35]. Authors found out that PEF treatment can improve extraction of carotenoids when 1.85 kV/cm was used, or it can lead to degradation of them when higher electric field (and hence higher energy input) of 3 kV/cm was applied. It is worth emphasizing that authors demonstrated also that there is a significant negative relationship between effectiveness of electroporation and carotenoid content, which can also explain the results obtained in currently presented research. The decrease effect of PEF treatment on carotenoids may be related with different phenomena. As aforementioned, carotenoids are sensitive for oxidation and PEF treatment may be accompanied by free radicals and reactive oxygen species production as a response for stress [36]. 

Combination of PEF and US led to degradation of carotenoids but, what is important, the level of effect depended on the sequence of US/PEF application. For instance, when US treatment was performed as a first step and afterwards bell pepper was treated by PEF at 1 kJ/kg carotenoids content reached 812 mg/100 g d.m. whereas when the sequence was reversed total carotenoid content was smaller by 17% and it was equal to 672 mg/100 g d.m. Similar results were found for the samples treated with PEF at 3 kJ/kg. The impact of sequence seems to be rather important and it is associated with mechanisms of sonication and PEF treatment. Since PEF treatment lead to rupture of cell membranes the leakage of intracellular content may appear. The application of US to such treated tissue most probably accelerates the mass transfer with surroundings and in consequence decreases carotenoids content. What more, free radicals and reactive oxygen species is also characteristic for ultrasound, which can also affect the carotenoid stability. In the literature, there are reports that indicate that plant tissue pre-treated by PEF and US or US and PEF and afterwards processed by drying is characterized by higher total carotenoid content. For instance, relative total carotenoid content of air dried carrots pre-treated by PEF followed by US was equal to 81% but when sequence was reversed (US followed by PEF) carotenoids retention was equal to 68% in comparison to fresh material [30]. These findings together with data reported in this paper imply that combined pre-treatment which is applied before processing may cause some other changes in processed food which are related with enzymes, responsible for pigments degradation, stability and activity or with microstructural changes that affect extractability.

### 2.2. Effect of Traditional and Non-Thermal Treatment on the Vitamin C of Red Bell Peppers

**Figure 2:**
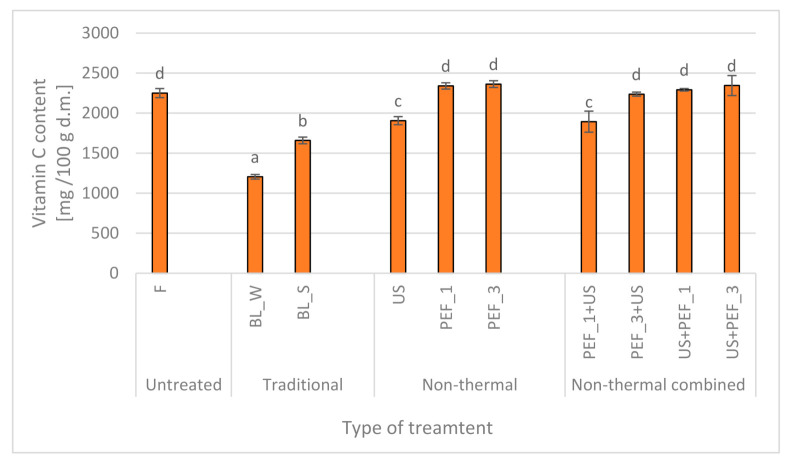
Vitamin C content in fresh (F) red bell peppers and subjected to traditional (blanching in water, BL_W, and blanching in steam, BL_S) and non-thermal single treatment (US—ultrasound, PEF_1 and PEF_3—pulsed electric field with energy input 1 and 3 kJ/kg, respectively) and combined treatment (PEF+ US and US + PEF), the same letters indicate homogeneous groups (Tukey’s HSD, *p* < 0.05), the error bars represent the standard deviation calculated from 3 repetitions.

The peppers are one of the sources of vitamin C. Fresh red bell pepper contain 88–277 mg/100 g fresh weight (f.w.) [6,37]. The vitamin C content in fresh red peppers and subjected to the pretreatment is presented in Figure 2. In fresh red bell peppers used for the study, the vitamin C content was 2250 mg/100 g d.m., which confirms that the peppers are a good source of ascorbic acid. This corresponded to 304 mg/100 g f.w. and was higher in comparison to value reported by Howard et al. [6]. It is well known, that vitamin C is heat-labile and its degradation in the product depends on the water activity, access of light, oxygen content and the presence of heavy metals (e.g., Cu, Fe), the type of raw material, its maturity, pretreatment and processing, especially at high temperatures [38,39]. Blanching caused a significant loss of vitamin C in peppers. Castro et al. [37] reported that higher degradation of ascorbic acid occurs with higher temperatures and longer treatment time. They observed 30% degradation of ascorbic acid in blanched red peppers. Similarly, the samples subjected to the blanching in water and stream resulted in vitamin C reduction around 46 and 26%, respectively. The vitamin C is water soluble, so blanching in water increases the leaching and dissolving ascorbic acid. The results obtained in the study are in line with the literature, which says that steam blanching preserves a higher amount of heat-sensitive compounds [17].

The use of the non-thermal technique for pretreatment such as ultrasound caused around 15% degradation of vitamin C in red peppers. Changes in the content of bioactive ingredients are the result of various phenomena that occur during sonication. Ultrasonic phenomena cause, among others, rupture of polymer chains and tissue cell walls, and as a result—better extraction of bioactive compounds from tissue treated with sound waves. At the same time, the formation of reactive oxygen forms as a result of cavitation can cause degradation of bioactive compounds. Due to the high-sensitivity of vitamin C and its solubility in water, the sonication effect on peppers was probably more linked with the long treatment in water (30 min) and formation of the reactive oxygen forms [20], especially when longer sonication is applied.

However, when non-thermal treatment as PEF was applied, regardless of the specific energy input, the vitamin C was similar or insignificantly higher (by 4 to 5%) in comparison to initial value. It is notated that PEF application may have no negative effect on thermolabile components in food [40]. Furthermore, PEF treatment, similarly to blanching, might cause inactivation of enzymes [41], which are responsible for ascorbic acid degradation [37]. PEF treatment can stimulate the stress reaction of the plant and led either to Reactive Oxygen Species (ROS) formation. What more, it can rise the production of secondary metabolites which are very often bioactive compounds. Another explanation is related with electroporation phenomenon and loss of the membrane continuity which increases extractability [20]. Similarly, the vitamin C content of the peppers was not affected by non-thermal combined treatment, with the exception of application of PEF_1 + US where 15% of the ascorbic acid reduction was observed, whereas all combined treated samples showed unchanged or slightly higher content of vitamin C content. Such results, which are unexpected at first sight confirm that effect of combined treatment is a superposition of many different processes and phenomena that occur during and after treatment. In this case, electroporated tissue was subjected to sonication which, as explained before for carotenoids, improved leakage of intracellular content. The lack of similar results for other variants of treatment most probably was related with the fact that leakage was compensated by processes that improve extractability of vitamin C from the tissue.

### 2.3. Effect of Traditional and Non-Thermal Treatment on the Polyphenols Content of Red Bell Peppers

**Figure 3:**
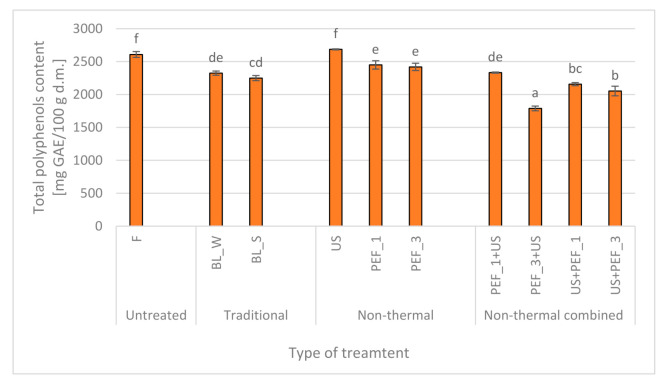
Total polyphenols content in fresh (F) red bell peppers and subjected to traditional (blanching in water, BL_W and blanching in steam, BL_S) and non-thermal single treatment (US—ultrasound, PEF_1 and PEF_3—pulsed electric field with energy input 1 and 3 kJ/kg, respectively) and combined treatment (PEF + US and US + PEF), the same letters indicate homogeneous groups (Tukey’s HSD, *p* < 0.05), the error bars represent the standard deviation calculated from 3 repetitions.

Chemical modifications that occur in food, which are related to OH groups and aromatic rings of polyphenols play an important role in their stability. The presence of oxygen, pH, temperature, metal ions, enzymes, ascorbic acid content, and others influence the stability of the polyphenols [42]. The polyphenols content of the red bell peppers treated with traditional (BL_W, BL_S) and non-thermal single (US, PEF) and combined treatment (PEF + US, US + PEF) is presented in Figure 3. Fruits and vegetables, including peppers, are a source of phenolics [5,43], and fresh material contained polyphenols in an amount equal 2607 ± 44 mg gallic acid (GAE)/100 g d.m. As mentioned by Deng et al. [42] temperature has a great impact on the polyphenols content in plant tissue. Due to this, in present study thermal treatment as steam blanching and blanching in water resulted in its reduction about 13.7 and 10.9%, respectively.

The ultrasound is used usually to improve extraction [44]. Therefore, sonication has an impact on the preservation of polyphenols in peppers tissue, which was slightly higher (*p* > 0.05) in comparison to initial polyphenols content in fresh peppers. Moreover, during this non-thermal treatment, which was conducted in water, the oxidation was limited [20]. When PEF was applied 6-7% decrease of the polyphenols content was noticed. The application of different specific energy inputs resulted in unchanged total polyphenols content in PEF treated peppers (Figure 3). As aforementioned utilization of PEF may cause inactivation of enzymes [45], however some studies indicate that it can also activate enzymes [46]. On the other hand, due to the plant stress caused by the PEF application the reactive oxygen species (ROS) can form [47]. Also, the used parameters of this non-thermal technics has an influence on the polyphenols content [35]. Wiktor et al. [35] noted that PEF treatment resulted in an increase, unchanging and decrease of total polyphenols content in apples depending on applied electric field intensity, pulsed number and specific energy input. These all effects might impact on polyphenols content also when the combined treatment was performed, where significant decrease of the TPC was noticed. What is also worth emphasizing non-thermal combined treatment caused similar or even higher reduction of total phenolic content compared to thermal treatment. Different patterns observed for total polyphenols and vitamin C, although both are polar and water soluble molecules, suggest that not only leaching of intracellular content plays important role but also enzymatic activity may be altered. Polyphenols liberated after PEF or US application are further oxidized most probably by polyphenols oxidase (PPO) during second step of treatment. The activity of PPO is in fact influenced by US or PEF as reported in the literature [48,49].

### 2.4. Effect of Traditional and Non-Thermal Treatment on the Antioxidant Activity of Red Bell Peppers

**Figure 4:**
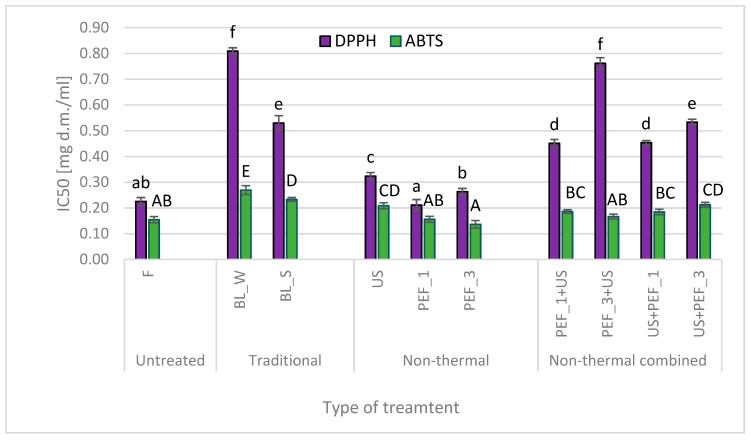
Antioxidant activity in fresh (F) red bell peppers and subjected to traditional (blanching in water, BL_W, and blanching in steam, BL_S) and non-thermal single treatment (US—ultrasound, PEF_1 and PE—pulsed electric field with energy input 1 and 3 kJ/kg, respectively) and combined treatment (PEF + US and US + PEF), the same letters indicate homogeneous groups (Tukey’s HSD, *p* < 0.05), the error bars represent the standard deviation calculated from 3 repetitions.

Antioxidant properties, measured by DPPH and ABTS assay and expressed as IC50, of bell pepper extracts are presented in Figure 4. Peppers contain carotenoids, flavonoids, polyphenols and vitamins C, which are responsible for antioxidant activity [3,4]. The highest antioxidant activity (the lowest IC50 value), measured by DPPH based method, was exhibited by fresh samples. All treatment increased values of IC50, however in the case of material pre-treated by PEF at 1 and 3 kJ/kg the change of antiradical activity was not relevant from statistical point of view. The biggest changes in free radical scavenging activity were found for bell pepper fruits subjected to thermal treatment. IC50 measured by DPPH for hot water blanched samples was equal to 0.81 mg d.m./mL and it was more than 3.5 higher than the value found for untreated, fresh material. Steam blanching also resulted in high reduction of antioxidant activity when compared to intact samples. As mentioned before, the smallest changes of antiradical properties were stated for bell pepper fruits treated by non-thermal methods. This is most probably related to higher availability of bioactive compounds that participate in antioxidant activity as discussed previously for phenolics, carotenoids and vitamin C. Antioxidant properties as assessed by DPPH assay of samples exposed for combined treatment were worse in comparison to material treated by PEF or US only and to fresh fruits. Most probably, it was related with long time of treatment (sonication lasted 30 min) which could result in degradation or reactions of some antioxidants. The effect of time is especially visible for sample marked as PEF_3 + US. This sample was treated with PEF at higher energy input level and afterwards, such electroporated sample with liberated bioactive molecules, were sonicated. Although, sonication was performed in water and oxygen availability was limited, the cavitation phenomenon caused free radical formation, improved stirring of intracellular content and thus intensified conditions of leakage or chemical reactions as it enhance mixing even at molecular level [50]. It is worth emphasizing that antiradical activity as expressed by DPPH assay were more differentiated than the results evaluated using ABTS method. It may be related with different sensitivity of assays on polarity of the bioactive compounds in the system [51]. Moreover, some studies indicate that DPPH is more suitable than ABTS assay for pigmented food, like bell peppers, that contains both hydrophobic and hydrophilic antioxidants [52]. Dorantes-Alvarez et al. [5] found that carotenoids and phenolics correlated with radical scavenging activity. In presented research significant (*p* < 0.05) correlation has been found between: IC50 measured by DPPH and ABTS (r = 0.652), IC50 measured by DPPH assay and total polyphenols content (r = −0.705), total carotenoid content (r = −0.861). In turn IC50 evaluated by ABTS method correlated significantly with vitamin C (r = −0.851) and total carotenoid content (r = −0.681).

### 2.5. Effect of Traditional and Non-Thermal Treatment on the Sugars Content of Red Bell Peppers

Table 1 presents sugars content in fresh red bell peppers and subjected to traditional (BL_W, BL_S) and non-thermal single (US, PEF) and combined treatment (PEF + US, US + PEF). In fresh peppers the sucrose, glucose and fructose were equal to 4.7 ± 0.1, 28.7 ± 0.5 and 29.7 ± 0.3 g/100 g d.m., respectively. Thermal treatment, when it was conducted in water resulted in the reduction of sugars content. This was linked with the injuring of the tissue and the mass transfer [53] where the sugars could easily move to the hot water. It has to be noted that the solubility of sugars in hot water was high even though the treatment lasted only 3 min. When steam blanching was used, slight loss of sugars was noticed. Nevertheless, changes in sucrose and fructose for peppers subjected to steam blanching was statistically significance in comparison to the intact sample.

For single non-thermal treatments, the sucrose content was not changed from statistical point of view, while alteration in glucose and fructose varied depending on the used treatment. The US treatment was performed in room temperature water, and for sonicated samples the glucose and fructose was significantly lower in comparison to untreated one. It means that loss of simple sugars was associated with a relatively long pre-treatment which lasted 30 min. During PEF treatment samples were immersed in room temperature water and treatment lasted very short (less than 1 min). Glucose content of PEF treated samples was similar or higher, when PEF with specific energy intake of 1 kJ/kg was applied, in comparison to fresh peppers. This means that PEF treatment might cause carbohydrates decomposition in a way that it can affect some other carbohydrates [54] or in improved extractability of sugars.

Combination of ultrasound with PEF treatment resulted in decrease of sugars content. On the one hand, the sugar dissolved in water, and on the other, carbohydrates hydrolysis could occur after PEF treatment as mentioned before. This is demonstrated by the higher content of simple sugars in combined treatment methods US + PEF compared to a single treatment only with ultrasound (US). At the same time, sugar content when single. However, a completely different trend was observed when the order of applied treatments was changed. For PEF + US combined treatment the sugar content was around 1.5 to 2 folds lower than in fresh peppers. At the beginning of treatment, the PEF caused permeabilization of the cells [26] of the red bell peppers tissue and then the sonication accelerated mass transfer [55]. Furthermore, there was not notice any significant influence of energy input PEF, when PEF + US was used. Also, reduction of the sugars was noticed in osmo-dehydrated cranberries subjected to the combined treatment as blanching with PEF and US [56]. The results show that this combination of treatment might be used to produce food with a reduced amount of sugars, which nowadays has a huge impact due to the rising group of people with overweight and obesity [57].

### 2.6. Cluster Analysis (CA)

**Figure 5:**
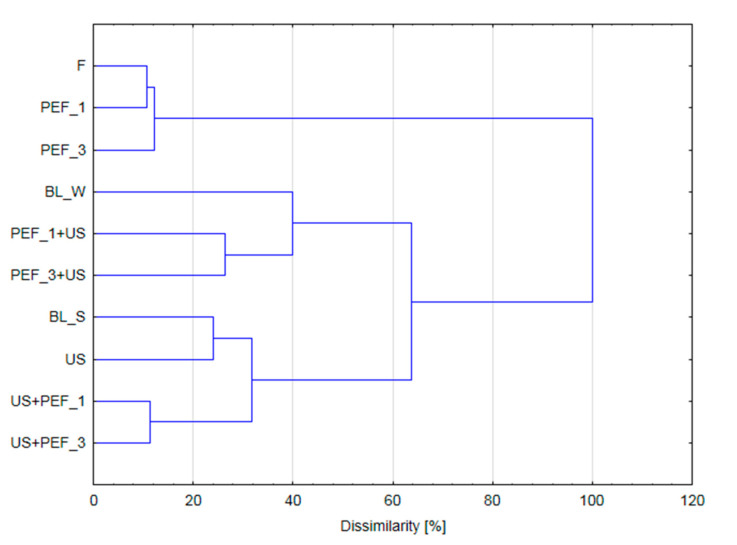
PCA for fresh (F) red bell pepper and subjected to traditional (blanching in water, BL_W, and blanching in steam, BL_S) and non-thermal single treatment (US—ultrasound, PEF_1 and PEF_3—pulsed electric field with energy input 1 and 3 kJ/kg, respectively) and combined treatment (PEF+ US and US + PEF).

Figure 5 presents results of Cluster Analysis that was performed including all investigated and normalized parameters. As it can be seen two big clusters can be identified. One group consist of three samples: fresh (F) and PEF pre-treated by 1 and 3 kJ/kg (PEF_1 and PEF_3, respectively). Another group agglomerated all other samples. However, within this second group, smaller clusters were identified. Interestingly, samples pretreated by PEF followed by US exhibited properties similar to samples blanched by hot water. In turn, bell peppers pretreated by ultrasound and US followed by PEF were similar to steam blanched samples. Such results indirectly confirm that application of US after PEF treatment intensifies leakage of bioactive compounds following similar mechanism that water blanching. 

## 3. Materials and Methods

### 3.1. Materials

The research material was red bell pepper (*Capsicum annuum* L.) purchased on the local market (Bronisze, Poland). Due to the seasonality of the raw material and ensuring repeatability of results, fruits from one batch were used. Material, before experiments, was stored in refrigerated conditions at temperature 4 ± 1 °C, humidity 85 ± 5%. Before the analysis, the fruits were washed by tap water and gently dried. The material was cut into pieces of 2 × 4 cm. The dry matter was determined according the AOAC (2002) by vacuum drying (Memmert VO400, Schwabach, Germany) (10 mPa, 70 °C, for 24 h) was 8.57 ± 0.23%.

### 3.2. Processing Procedure

#### 3.2.1. Blanching with Water (BL_W) and Steam (BL_S) 

Blanching with water and steam was performed using tap water. The chopped material was placed directly in a boiling water bath with a water temperature of 98 °C. The ratio of water to material was 2:1. Blanching with steam was carried out on a sieve placed above the evaporating water at a temperature of 98 °C. During the process, the bed of material was mixed every 15 s. The processes were carried out for 3 min. After treatment, the samples were strained on a sieve, cooled in a stream of cold water (15 s), and the remaining water was removed on the filter paper. The treatment was repeated three times.

#### 3.2.2. Ultrasonic Treatment (US) 

Ultrasound application was carried out using the immersion method. The material was placed in an ultrasonic bath (MKD-3, MKD Ultrasonics, Warsaw, Poland) filled with water at a temperature of 20.2 ± 1.3 °C. The ultrasonic frequency was 21 kHz and the power generated by sonotrodes was 300 W. The ratio of tap water to material was 4:1. The sonication process lasted 30 min, then the samples were filtered on a sieve and dried on filter paper. The treatment was repeated three times.

#### 3.2.3. Pulsed Electric Field Treatment (PEF)

PEF application was carried out using a batch pilot scale PEF unit (PEF PilotTM, Elea GmbH, Quakenbrück, Germany). The device generated voltage up to 30 kV and provided monopolar-exponential decay pulses with pulse width of 40 ms. The frequency of pulses application was set up to 2 Hz. The gap between the stainless-steel electrodes in the treatment chamber was 280 mm. Bell peppers were weighed and placed inside the treatment chamber. Afterwards, potable water (σ = 220 μS/cm and T = 21 ± 1 °C) was added in order to immerse material in conductive medium. The total mass of the bell pepper was 200 ± 5 g and the product to water ratio was 1:24, respectively. Specific energy intake (kJ/kg) was adjusted by adapting the number of pulses. The trials were done by applying specific energies within a range of 1–3 kJ/kg and a field strength of 1.07 kV/cm. The specific energy intake W*_spec_* (kJ/kg) and electric field strength E (kV/cm) were calculated according to the following Equations (1) and (2).

W*_spec_* = (U^2^·C·n)/2·m (1)

E = U/d(2)

where n is the number of pulses (-); m is the mass of the treated samples (kg); U is the voltage (kV) and d is the distance between electrodes (cm); C–is the capacitance (F).

PEF pretreatment was performed 3 times for each investigated specific energy input. PEF pre-treatment conditions were selected based on the effectiveness of electroporation as assessed by electrical conductivity measurements which was described in details [58].

#### 3.2.4. Combined Methods

The combination of the traditional and non-thermal treatment was studied. All combination of the treatment was presented in Table 2. 

### 3.3. Chemical Analysis

#### 3.3.1. Total Carotenoids Content (TCC)

The total carotenoids content was determined based on the spectrophotometric method described in the Polish Standard PN-EN 12136:2000 [59]. To 0.7 g (m_1_) homogenized sample 20 mL of distilled water, 1 mL Carrez I soltion, 1 mL Carrez II solution (VWR Chemicals BDH Prolabo, Leuven, Belgium) were added and the mixture was mixed 2 min on a vortex magnetic stirrer. The solution was centrifuged with acceleration of 2000 g for 5 min. The colorless supernatant was decanted and 25 mL of acetone was added to the precipitate, mixed on a vortex (3 min) and centrifuged. The acetone solution was decanted into a laboratory separator, 25 mL of petroleum ether was added, the mixture was shaken thoroughly and allowed to separate the phases. The colorless phase was discarded. The sediment was extracted again with 25 mL of acetone. Another portion of the supernatant was combined with the liquid in a separatory funnel, 20 mL of petroleum ether, 10 mL of distilled water were added, shaken and the lower acetone phase removed. The ether phase was poured into centrifuge tubes with 1.5 g of anhydrous sodium sulfate and centrifuged. The supernatant solution was poured into a 100 mL flask (m_2_) and filled up to 100 g with reagent. The absorbance of the solutions was measured at 450 nm (Spectronic 200; Thermo Fisher Scientific Inc., Waltham, MA, USA). The total carotenoids content was calculated from the formula (3):

ρ (_C40H56_) = A_450_ × 4.00 × (m_2_/m_1_) × 10 (3)

where:

ρ (_C40H56_)–total carotenoids, in mg/100 g
A_450_–absorbance of the petroleum ether extract
4.00–average in the conversion factor determined on the basis of the ring test, taking into account the average β-carotene absorption coefficient in petroleum ether and dilutions made during the analysis.

#### 3.3.2. Vitamin C

The UPLC-PDA system (WATERS Acquity H-Class, Milford, MA, USA) was used to detected L-ascorbic acid [60]. 0.5 g grounded (IKA A11 basic, IKA- Labortechnik, Staufen, Germany) pepper was mixed with 35 mL extraction solution (3%meta-phosphoric acid, 8% acetic acid, 1 mM EDTA—VWR Chemicals BDH Prolabo, Leuven, Belgium), 10 min vortexed and centrifuged (5 min, 6000 rpm). Supernatant was filtered through 0.22 μm polytetrafluoroethylene (PTFE) syringe filters. 1 mL solution was added to 1 mL eluent and injected (5 µL). Separation was performed using an WATERS Acquity UPLC HSS T3 (2.1 × 100 mm, 1.8 μm; Waters, Ireland) with pre-column BEH C18 (2.1 × 5 mm, 1.7 µm; Waters, Ireland). Mobile phase (Milli-Q water with 0.1% (*v*/*v*) formic acid) flow was 0.25 mL/min. Column thermostat temperature was 25 °C and the samples were kept at 4 °C. The spectrum at wavelength 245 nm was analyzed. Vitamin C content was calculated in relation to the calibration curve prepared for the L-ascorbic acid (VWR Chemicals BDH Prolabo, Leuven, Belgium) analytical standard (0.005–0.100 mg/mL). The analysis was performed in triplicate.

#### 3.3.3. Total Polyphenols Content (TPC)

The total polyphenol content was determined by the Folin-Ciocalteu spectrophotometric method [56] based on the colorful reaction of molybdenum reduction by phenolic compounds in alkaline environment. The grounded (IKA A11 basic, IKA- Labortechnik, Staufen, Germany) samples were extracted with a solution of 80% ethyl alcohol and filtered through a filter paper. 0.3 mL of Folin-Ciocalteu (Merck, Darmstadt, Germany) solution was added to 0.18 mL of extract and 4.92 mL distilled water. The contents of the tubes were mixed and after 3 min 0.6 mL of 17.7% sodium carbonate solution was added. After 60 min of incubation in the dark, absorbance at 750 nm was measured with a spectrophotometer (Spectronic 200; Thermo Fisher Scientific Inc., Waltham, MA, USA) against a blank consisting of all reagents and water instead of extract. In order to determine the amount of polyphenolic compounds, a calibration curve for gallic acid was prepared in the range: 1–5 mg/mL. The analysis was performed in triplicate.

#### 3.3.4. Antioxidant Activity (AA)

Antioxidant activity was determined based on the degree of inhibition of the synthetic 2,2-diphenyl-1-picrylhydrazyl radical (DPPH^•^) and 2,2-azinobis(3-ethylbenzothiazoline-6-sulfonate) cation-radical (ABTS^•+^) by antioxidants extracted from pepper samples [39,53]. The analysis was performed in triplicate.

##### *DPPH assay* 

A radical stock solution was prepared by dissolving 25 mg DPPH (Sigma-Aldrich, Steinheim, Germany) in 100 mL methanol. Before the analysis, the solution was diluted with 80% alcohol to obtain absorbance at 515 nm in the 1.360–1.420 range. To the known volume of the alcohol extract of pepper samples (prepared for the determination of polyphenols), 2 mL of radical solution was added, mixed and incubated at room temperature in the absence of light. After 30 min, absorbance at 515 nm was measured. The antioxidant capacity was expressed as IC50–concentration required to obtain a 50% antioxidant effect.

##### *ABTS assay* 

To generate ABTS free radicals to 0.0384 g of 2,2-azinobis (3-ethylbenzothiazoline-6-sulfonate) (Sigma-Aldrich, Steinheim, Germany) cation-radical, 0.0066 g K_2_S_2_O_8_ (Sigma-Aldrich, Steinheim, Germany) was added and made up with distilled water to a volume of 10 mL. The solution was mixed and placed in the fridge for 16 h. The working solution was prepared by diluting the stock solution with a 80% ethyl alcohol solution of the concentrated radical solution to obtain absorbance at a wavelength of 734 nm within 0.680–0.720. 2 mL of radical solution was added to the tubes with different extract concentrations (20–80 µL). Mixed on a vortex and incubated at room temperature in the dark. After 6 min, the absorbance of the solutions and the radical were measured. The antioxidant capacity was expressed as IC50–concentration required to obtain a 50% antioxidant effect.

#### 3.3.5. Sugars Content (SC)

The liquid chromatography method with refractive index detection was used to determine sugars [61]. The system was equipped with a quaternary pump (Waters 515, Milford, MA, USA), autosampler (Waters 717, Milford, MA, USA), column thermostat and RI detector (Waters 2414, Milford, MA, USA). The separation was carried out using a 300 × 6.5 mm Waters Sugar Pak I column with a Sugar-Pak guard column. The grounded material was extracted with distilled water at 80 °C for 4 h. The solution was filtered through a 0.22 μm PTFE syringe filter and was dosed into the system. The injection volume was 10 µL. The analysis was carried out under isocratic conditions, the flow rate of the mobile phase (redistilled Milli-Q water) was 0.6 mL/min, the column temperature was 90 °C and detector temperature was 50 °C. The quantitative analysis was carried out based on the prepared calibration curves for sucrose, glucose and fructose standards (Sigma-Aldrich, Steinheim, Germany). The analysis was performed in duplicate.

### 3.4. Statistical Analysis

To assess significant differences between investigated samples the ANOVA procedure (at *p* < 0.05) was applied using the Tukey test. Moreover, the Cluster Analysis (CA) was performed taking into account all of investigated variables. The statistical analysis was conducted using STATISTICA 13 software (TIBCO Software, Palo Alto, CA, USA).

## 4. Conclusions

The use of thermal operations has reduced the content of vitamin C, carotenoids, polyphenols, and antioxidant activity. However, within thermal methods, higher preservation of bioactive compounds was stated for steam blanched material as compared to blanching in water. When the non-thermal, unconventional methods as sonication (US) and pulsed electric field treatment (PEF) and their combinations were used, comparable or lower contents of bioactive compounds were obtained in comparison to fresh pepper. The sonication in most cases caused the reduction of bioactive compounds (with the exception of total phenolic content), which was probably related with the long treatment time (30 min). Also, the configuration of the non-thermal combined treatment had an impact on plant tissue. Better retention of vitamin C and carotenoids content was observed for the treatment combination based on sonication with a pulsed electric field the sequence of US + PEF than PEF + US. Selection of appropriate operations and parameters let to obtain products characterized by similar or slightly lower content of high bioactive compound in comparison to fresh red bell pepper. Moreover, when the combined treatment based on a pulsed electric field with sonication (PEF + US) was applied, regardless of the used parameters of PEF (1 or 3 kJ/kg), the significantly lower sugars content was noticed in comparison to other treatments. This indicates the possibility of using this type of treatment to obtain products with reduced sugars content, which is very important due to the growing problem of obesity present in society [57].

## Figures and Tables

**Table 1:** Sugars content in fresh (F) red bell peppers and subjected to traditional (blanching in water BL_W and blanching in steam BL_S) and non-thermal single treatment (US–ultrasound, PEF_1 and PEF_3–pulsed electric field with energy input 1 and 3 kJ/kg, respectively) and combined treatment (PEF + US and US + PEF), error bars indicate ± SD (standard deviation calculated from 2 repetitions).

Type of Treatment	Sugars Content		
Single Treatment	Sucrose [g/100 g d.m.]	Glucose [g/100 g d.m.]	Fructose [g/100 g d.m.]
F	4.7 ± 0.1 d	28.7 ± 0.5 d	29.7 ± 0.3 d
BL_W	3.1 ± 0.2 b	17.3 ± 1.4 a	20.7 ± 0.6 a
BL_S	4.1 ± 0.1 c	27.0 ± 0.8 cd	26.2 ± 0.7 c
US	4.2 ± 0.1 cd	21.5 ± 0.5 b	23.4 ± 0.7 b
PEF_1	4.6 ± 0.3 cd	31.8 ± 0.3 e	30.6 ± 0.9 d
PEF_3	4.2 ± 0.1 cd	29.3 ± 0.4 d	27.3 ± 1.3 c
**Combined treatment**			
PEF_1 + US	2.4 ± 0.3 a	16.2 ± 1.1 a	19.6 ± 0.7 a
PEF_3 + US	2.2 ± 0.2 a	18.4 ± 1.2 a	20.1 ± 0.4 a
US + PEF_1	4.6 ± 0.2 cd	24.7 ± 0.5 c	23.6 ± 0.5 b
US + PEF_3	4.3 ± 0.1 cd	26.2 ± 1.0 c	25.7 ± 0.6 c

The same letters in columns indicate homogeneous groups (Tukey’s HSD, *p* < 0.05).

**Table 2:** Abbreviations and parameters of conducted treatment for red bell pepper.

Treatment	Description	Parameters of Treatment
F	untreated sample	-
BL_W	blanching in water	temp: 98 °C, time: 3 min
BL_S	blanching in steam	temp: 98 °C, time: 3 min
US	ultrasound treatment	ultrasound intensity 3 W/cm^2^, frequency: 21 kHz, time: 30 min
PEF_1	pulsed electric field treatment	pulse number: 6, electric field intensity: 1.07 kV/cm, ^1^ W_s_: 1 kJ/kg
PEF_3	pulsed electric field treatment	pulse number: 12, electric field intensity: 1.07 kV/cm, ^1^ W_s_: 3 kJ/kg
**Combined treatment**	**I step**	**II step**
	**Parameters of treatment**	
PEF_1 + US	pulsed electric field pulse number: 12, electric field intensity: 1.07 kV/cm, ^1^ W_s_: 1 kJ/kg	ultrasound treatment, ultrasound intensity 3 W/cm^2^, frequency: 21 kHz, time: 30 min
PEF_3 + US	pulsed electric field pulse number: 34, electric field intensity: 1.07 kV/cm, ^1^ W_s_: 3 kJ/kg	ultrasound treatment, ultrasound intensity 3 W/cm^2^, frequency: 21 kHz, time: 30 min
US + PEF_1	ultrasound treatment, ultrasound intensity 3 W/cm^2^, frequency: 21 kHz, time: 30 min	pulsed electric field pulse number: 12, electric field intensity: 1.07 kV/cm, ^1^ W_s_: 1 kJ/kg
US + PEF_3	ultrasound treatment, ultrasound intensity 3 W/cm^2^, frequency: 21 kHz, time: 30 min	pulsed electric field pulse number: 34, electric field intensity: 1.07 kV/cm, ^1^ W_s_: 3 kJ/kg

^1^ W_s_ is specific energy intake.
